# NEUROPATHOLOGY AND GAIT SPEED DECLINE IN OLDER ADULTS: THE ATHEROSCLEROSIS RISK IN COMMUNITIES (ARIC) STUDY

**DOI:** 10.1093/geroni/igz038.3377

**Published:** 2019-11-08

**Authors:** Kevin J Sullivan, Michael Griswold, Timothy Hughes, Christina E Hugenschmidt, Samuel Lockhart, Thomas Mosley, Rebecca Gottesman, Gwen Windham

**Affiliations:** 1 University of Mississippi Medical Center MIND Center, Jackson, Mississippi, United States; 2 Wake Forest School of Medicine, Winston-Salem, North Carolina, United States; 3 Johns Hopkins University, Baltimore, Maryland, United States

## Abstract

Neuropathological markers including amyloid-beta (Aβ) have been implicated in mobility decline in older adults, but no studies have examined the relationship between these markers and longitudinal change in gait speed in a racially diverse community-based sample. In the multi-site prospective ARIC study, a subsample of participants (n=1,978, mean age=76.3, 28.5% black) underwent brain MRI at Visit 5 (2011-13). Of these, 343 participants (mean age=75.9, 42.6% black) completed PET scans using the tracer florbetapir to estimate global brain Aβ. We investigated the relationship between four neuropathological markers [white matter hyperintensities (WMH; log2cm3), infarcts (present/absent), brain atrophy (log2cm3), and global Aβ (log2SUVR)] with cross-sectional usual pace gait speed (cm/s) over 4 meters, and change in gait speed through Visits 6 (2016-17) and 7 (2018-19). Linear regression models were adjusted for age, site, sex, education, BMI, intracranial volume, and all race interactions. Cross-sectionally, slower gait was associated with higher WMH volume (β=-2.16, 95%CI: -2.92, -1.39), infarcts (β=-5.81, 95%CI: -7.86, -3.76), and brain atrophy (β=-16.39, 95%CI: -21.07, -11.71). Longitudinally, only higher WMH volume was statistically associated with gait speed decline (β=-0.14, 95%CI: -0.28, -0.01). Global Aβ was not statistically associated with gait speed cross-sectionally (β=-.269, 95%CI: -8.11, 7.57) or longitudinally (β=-1.16, 95%CI: -2.94, 0.62). There were no significant interactions with race. Detrimental relations of cerebral small vessel disease to mobility and mobility decline were observed across race in this diverse sample. The magnitude of the Aβ association with gait speed decline was high, although not statistically significant in the smaller PET subsample.

